# Prevalence of Central Compartment Lymph Node Metastases in Papillary Thyroid Micro-Carcinoma: A Retrospective Evaluation of Predictive Preoperative Features

**DOI:** 10.3390/cancers13236028

**Published:** 2021-11-30

**Authors:** Marta Tagliabue, Gioacchino Giugliano, Maria Cecilia Mariani, Manila Rubino, Enrica Grosso, Francesco Chu, Anna Calastri, Fausto Antonio Maffini, Giovanni Mauri, Elvio De Fiori, Marco Federico Manzoni, Mohssen Ansarin

**Affiliations:** 1 Division of Otolaryngology and Head and Neck Surgery, European Institute of Oncology (IEO) IRCCS, 20141 Milan, Italy; marta.tagliabue@ieo.it (M.T.); gioacchino.giugliano@ieo.it (G.G.); mariacecilia.mariani@ieo.it (M.C.M.); enrica.grosso@ieo.it (E.G.); francesco.chu@ieo.it (F.C.); marcofederico.manzoni@ieo.it (M.F.M.); mohssen.ansarin@ieo.it (M.A.); 2 Department of Biomedical Sciences, University of Sassari, 07100 Sassari, Italy; 3 Division of Gastrointestinal Medical Oncology and Neuroendocrine Tumors, European Institute of Oncology (IEO) IRCCS, 20141 Milan, Italy; manila.rubino@ieo.it; 4 Department of Otorhinolaryngology, Fondazione IRCCS, Policlinico San Matteo, 27100 Pavia, Italy; anna.calastri01@universitadipavia.it; 5 Division of Pathology, European Institute of Oncology (IEO) IRCCS, 20141 Milan, Italy; fausto.maffini@ieo.it; 6 Department of Oncology and Hematology-Oncology, University of Milan, 20122 Milan, Italy; 7 Division of Interventional Radiology, European Institute of Oncology (IEO) IRCCS, 20141 Milan, Italy; 8 Department of Radiology, European Institute of Oncology (IEO) IRCCS, 20141 Milan, Italy; elvio.defiori@ieo.it; 9 Institute of Endocrine and Metabolic Sciences, San Raffaele Hospital, Vita-Salute San Raffaele University, 20132 Milan, Italy

**Keywords:** papillary thyroid carcinomas, central lymph node metastases, thyroid micro-carcinoma, prophylactic central neck dissection

## Abstract

**Simple Summary:**

The present study focused on patients affected by stage pT1a papillary thyroid micro-carcinomas that were treated with surgery and central lymph node dissection. In this study, male sex, low age, and sub-capsular carcinoma localization resulted as independent predictive factors for central lymph node metastases.

**Abstract:**

Papillary thyroid micro-carcinomas are considered relatively indolent carcinomas, often occult and incidental, with good prognosis and favorable outcomes. Despite these findings, central lymph node metastases are common, and are related to a poor prognosis for the patient. We performed a retrospective analysis on patients treated with surgery for stage pT1a papillary thyroid micro-carcinomas. One hundred ninety-five patients were included in the analyses. The presence of central lymph node metastases was identified and studied. A multivariate analysis employing binary logistic regression was used to calculate adjusted odds ratios with 95% confidence intervals of possible central lymph node metastases risk factors. In the performed multivariate analysis, male gender, younger age, and histopathological characteristics, such as a tumor sub-capsular localization, were significantly associated with central lymph node metastases in pT1a patients. Central compartment lymph node metastases are present in a non-negligible number of cases in patients with papillary thyroid micro-carcinoma undergoing surgical resection. Studying these factors could be an effective tool for predicting patients’ central lymph node metastases in papillary thyroid micro-carcinomas, defining a tailored surgical treatment in the future.

## 1. Introduction

Papillary thyroid micro-carcinoma (PTMC) is defined by the World Health Organization as a papillary carcinoma (PTC) with a maximum size less than or equal to 1 cm [1]. In recent decades, the incidence of PTC has been increasing, and PTMCs in particular have been increasingly detected. The higher incidence could be partly due to increased diagnosis determined by improvements in imaging techniques and cytological examinations, while possible effects of lifestyles cannot be excluded, such as the salt iodization program [2,3,4]. Despite this increase, most PTMC patients have an indolent clinical course and a favorable prognosis. Unfortunately, a non-negligible number of patients with recurrence and aggressive disease have been found in the literature [5,6,7,8,9,10,11,12]. At the time of surgery, central lymph node metastases (CLNM) were observed in 20% to 90% of PTMC patients in different studies, despite negative preoperative imaging evaluation [13,14,15,16,17,18,19]. While the clinical significance of PTMC lymph node metastases remains a matter of discussion, recent studies have reported CLNM as a potential marker of aggressive behavior of PTMC and a risk factor for recurrence, distant metastases, reduced survival, and higher morbidity [20,21,22,23,24].

To date, there is no consensus on the appropriate therapy strategy for PTMC patients. In the literature, the recommendation varies from an observational approach alone, to ultrasound-guided minimally invasive treatments, to conservative surgical treatment, and even to radical thyroidectomy with or without prophylactic central neck dissection [10,12,23,24,25,26,27,28,29,30,31,32,33,34]. Almost all clinicians agreed CLND should be performed in the case of preoperative evidence of CLNM. Hence, an accurate evaluation of the presence of CLNM is very important for choosing the central lymph node dissection for PTMC surgery in thyroid cancer patients.

The present study aims to evaluate the incidence of CLNM in a large series of patients affected by p-T1a PTMC, treated at a single institution over 23 years, and to identify possible risk factors associated with the presence of CLNM.

## 2. Materials and Methods

A retrospective cohort study at a single institution was conducted. Between January 1995 and January 2018, 3013 patients underwent thyroidectomy at the European Institute of Oncology, IRCCS—Thyroid Unit, Milan, Italy (IEO). A total of 2818 patients were excluded for: final benign disease, history of a previous thyroidectomy, age < 18 years, non-PTC carcinoma (follicular/medullary/anaplastic), mixed-type thyroid carcinoma [35], tumor size > 1 cm, and pT1b–pT4 stage.

All the patients were staged according to the TNM seventh edition [36].

One hundred ninety-five pT1a PTMC patients with available and adequately detailed medical records were included in the analyses. All the included patients underwent thyroid surgery plus prophylactic or therapeutic standardized-anatomical central lymph node dissection [37].

All patients were evaluated with a complete clinical examination and neck ultrasonography (US). US was performed to estimate the size of the lesion, its intra/extrathyroidal extension, and the presence of suspected cervical-lymph node metastases (LNM). Fine-needle aspiration cytology (FNAC) analysis was also performed on the thyroid nodule. A thyroid lobectomy was performed in cases with no evidence of extrathyroid extension (ETE), underlined at the preoperative US, and monolateral carcinoma. Total thyroidectomy was performed in cases with a bilateral carcinoma, familiar history of thyroid cancer, presence of ETE at the preoperative US, that according to the TNM seventh edition were classified as cT3 tumors, thyroiditis, and uncertain contralateral nodules. The final decision about the extent of surgery was determined by a decision-making discussion in a multidisciplinary team and with patient consensus.

According to the standardized-anatomical IEO surgical procedure [37], prophylactic central neck dissection was performed on clinically node-negative patients with macroscopic/microscopic ETE if the nodule FNAC was Tyr 3, 4, or 5 (classification of thyroid carcinoma risk stratification for FNAC following the ATA guidelines), or according to the surgeon and multidisciplinary team decision [30,38].

The “Tyr 3, 4, or 5” definition follows the Italian classification guidelines for the cytological alterations present in the thyroid FNAC. Tyr 3 is comparable to the classification Bethesda 3 and 4, Tyr 4 to Bethesda 5, and Tyr 5 to Bethesda 6. Increasing the category increases the risk of thyroid cancer [30,39,40]. Therefore, all patients with cytology greater than or equal to Tyr 3 or Bethesda 3 underwent prophylactic central neck dissection.

Therapeutic central neck dissection was performed if suspicious CLNM were detected preoperatively by the US or during surgery [38].

Collected data on clinical patients’ features were: gender, age at diagnosis, histopathological parameters including location in the thyroid gland (parenchymal vs. sub-capsular), multifocality (multifocal vs. unifocal), histological variants of PTC (follicular vs. classic) [41], and the presence of central compartment lymph node metastases.

The postoperative surgery complications collected were permanent hypocalcemia (defined as hypocalcemia that persists for more than 12 months after surgery) and recurrent laryngeal nerve paralysis [42].

Recurrence risk stratification was evaluated according to ATA guidelines [30].

The statistical analysis was conducted with SPSS version 23.0 (SPSS Inc., Chicago, IL, USA). Categorical variables were indicated as frequency, parametric continuous variables were indicated as means ± standard deviation (SD) (*p* > 0.05 in Kolmogorov–Smirnov test), and non-parametric variables were expressed as medians with an interquartile range (IQR). Differences in variable frequencies between groups were calculated using Fisher’s exact test, independent sample Student’s *t*-test for parametric continuous variables, and Mann–Whitney U test for non-parametric continuous variables.

A multivariate analysis employing binary logistic regression was used to calculate adjusted odds ratios (ORs) with 95% confidence intervals (CIs) of possible CLNM risk factors. All statistical tests were two-sided.

Institutional review board approval was achieved, and patients’ informed consent was waived (IEO cod IEO2044).

## 3. Results

The baseline and clinicopathological characteristics of the included 195 patients are summarized in Table 1. The mean age was 48 ± 13 (standard deviation) years, and there were 163 women (83.6%) and 32 men (16.4%). One hundred thirty-nine patients (71.3%) underwent total-thyroidectomy, while lobectomy was performed in 56 (28.7%). All patients included in this analysis underwent bilateral or unilateral central lymph node dissection for prophylactic or therapeutic purposes. A multifocal tumor was found in 56 patients (28.7%), sub-capsular localization was diagnosed in 66 patients (33.8%), and 52 PTMC (26.7%) were a follicular variant subtype. The central metastases percentage for patients undergoing total thyroidectomy vs. lobectomy was comparable: 23% vs. 28%, respectively.

Five patients out of one hundred ninety-five (2.5%) presented a capsular contact with possible microscopic infiltration on the pre-operative ultrasound. In one patient, the final histopathological examination showed “without sure signs of extracapsular extension”, for which it was staged as pT1a according to the nodule dimension.

Thirty-seven (18%) patients underwent iodine therapy, according to their final pathological stage.

The median follow-up was 5.4 years (0–17.05). None of the 195 patients died of thyroid cancer, and one had local neck recurrence that was treated surgically and with iodine 131 then recovered at the last follow-up.

The lymph nodes were negative in 143 patients (73.3%), and CLNM were found in 52 patients (26.7%). An average of 5.1 (0–47) lymph nodes were removed, of which 8% were metastatic lymph nodes and 92% were healthy. In the group of patients with pN1, an average of 11 lymph nodes, with a range of 1–47, were removed, the mean number of metastatic lymph nodes was 2.2 (1–12), and 8.9 (0–46) were healthy.

Of the 52 patients with metastases, 3 (5%) had massive metastases (>10 mm), 22 (42%) had micro-metastases (<0.2 mm), and 72 patients (51%) had metastases of ≥0.2 and ≤10. The mean metastases size was 3.3 mm (range 0.2–11 mm). Three patients had large deposits (>1 cm) with extra-nodal extension, and the average ratio of involved/uninvolved nodes was 0.13 on the entire study population and 0.48 for patients with CLNM.

The clinical and pathological characteristics of the CLNM positive patient group and CLNM negative group are summarized in Table 2.

There were no significant differences in the rate of multifocality and histopathological subtype of PTMC (*p* = 0.07 and *p* = 0.2, respectively). The significant differences between the two groups concern sex, localization of the tumor, and age. In particular, lymph node metastases were found to be more frequent in male patients than in females (46.9% vs. 22.7%, *p* = 0.008), in sub-capsular lesions compared to parenchymal ones (37.8% vs. 20.9%, *p* = 0.01), and in younger patients (44.7 ± 13.5 vs. 50.3 ± 12.3, *p* = 0.012).

Regarding post-operative complications, 67 (34%) patients reported transient hypocalcemia and 7 (3%) persistent hypocalcemia. Recurrent laryngeal nerve paralysis was recorded as transient in 12 (6%) patients, and no permanent recurrent laryngeal nerve paralysis occurred.

Post-operative thyroglobulin levels were on average 1.06 (0–20) ng/mL.

The multivariate logistic regression analysis showed that male sex, sub-capsular carcinoma localization, and younger age were statistically significant in predicting CLNM in PTMC (Table 3).

## 4. Discussion

The present study focused on patients affected by stage pT1a PTMC treated with surgery and CLN dissection. In this study male sex, low age, and sub-capsular carcinoma localization resulted as independent predictive factors for CLNM. In the analyzed cohort, CLNM were found in 52 patients (26.7%).

Different studies have reported that PTMC with ETE has an increased risk of CLNM [43,44,45]. Additionally, sub-capsular localization, defined as a carcinoma abutting on the thyroid capsule at histopathological evaluation, might be associated with an increased risk of CLNM, and sub-capsular localization at US imaging might represent a preoperative imaging biomarker indicative of risk of ETE and possible risk factors for CLNM [46].

Siddiqui et al. published an article in 2016 in which ETE was a significant predictor in the multivariate analysis of CLNM with an OR of 4.46 (OR of 16 for lateral neck metastases) [44]. In our work, according to the inclusion criteria, patients with ETE were excluded because, according to the TNM seventh edition, they were staged as pT3 tumors. Furthermore, 2.5% of patients had capsular contact and possible microscopic infiltration on preoperative ultrasound. However, this was not statistically significant in our sample.

In previous studies, the male gender has been suggested as an important risk factor for CLNM [47,48]. In the present work, the CLNM positive rate in males was 46.9% (vs. 22.7% in female patients). This result was confirmed by the multivariate analysis that showed male sex as an independent predictive factor of CLNM (*p* = 0.014).

In addition, age has been shown to be a risk factor [48,49,50]. Our study observed that younger age was a significant risk factor for CLNM. This is in agreement with different published studies and meta-analyses, even if in our analysis the standard deviation is high and the value is not far from the mean [48,49,50].

Multifocality is generally considered a risk factor for lymph node metastases. Still, there is a lack of evidence-based data to confirm this relationship, and it is reported in approximately 20–40% of the PTMCs [9,10,51,52]. Multifocal disease was present in 28.7% of the patients, and its presence was not statistically significant for CLNM.

The incidence of PTMC has been steadily increasing within recent years, primarily due to the extensive use of thyroid ultrasonography and an improved rate of detection, as well as an effect of environmental lifestyle changes [2,3,8,53,54,55,56].

PTMCs are considered relatively indolent carcinomas, often occult and incidental, with good prognosis and favorable outcomes [8,9,10,11,12,22]. Despite these findings, CLNM are common, with an incidence between 20% and 90%, and most of these are less than 5 mm [13,14,15,16,17,18,19,57]. Unfortunately, the sensitivity of current ultrasound methods for detecting CLNM is very low, ranging from 23% to 53.2%, and just 40% of the nodes are ≥5 mm [58,59]. Although some studies showed that these metastases did not affect survival, most authors underlined that regional LNM (CLNM) are associated with increased local recurrence rates and reduced survival [60,61].

One publication reported that a central compartment dissection may decrease the false-positive detection of skip metastases in PTC. Moreover, in the case of central nodal recurrence, re-operative dissection can increase complications rates; therefore, a one-step surgery should be preferred [62].

According to some work, adequate lymphadenectomy could be a better choice to ensure no residual metastatic lymph nodes and reduce secondary surgery rates [24].

Furthermore, performing a prophylactic central dissection in a center with a large flow of patients, in selected cases such as those studied in our series, is not associated with an increased risk of complications. Several recent meta-analyses underline how it is associated with a lower number of local relapses [63,64]. However, this position remains contested; some authors report the importance of different approaches, such as radioactive iodine ablation, in low-risk cases and cite insufficient evidence for routinely performing central neck dissection to improve patients’ prognosis [65,66]. In our study, all patients presented excellent survival. Local relapse in the neck occurred in one patient with metastases of the central compartment, and postoperative complications were very low [67]. 

Since there are few permanent surgical complications, we suggest recurrent dissection remains an effective measure to better know the patient’s disease and treatment, determining the real tumor stage, other postoperative treatments, and prognosis with greater accuracy.

To date, standardized and accepted international guidelines for the management of PTMCs, with or without CLNM, have not yet been established. In most cases, only a less-radical surgical treatment, ultrasound-guided minimally invasive treatments, or an observational approach alone is recommended. Some authors have underlined that observation alone or surgery with conservative treatment (lobectomy) should be performed for low-risk tumors if they show neither unfavorable features nor the clinical suspicion of nodal metastases [12,29,68]. Recently, in some centers, minimally invasive treatments have been successfully used in the treatment of PTMCs as an alternative to active surveillance, in cases with no suspected LNM and no evidence of capsular involvement [27,69].

Considering that the high frequency of CLNM in cN0 PTMC cannot be ignored, it remains a controversial matter whether prophylactic central lymph node dissection should be performed [60,70,71,72]. The American Thyroid Association (ATA) 2016 guidelines recommend prophylactic dissection in PTC patients with cN1, but it remains debated in patients with cN0 [30]. This restriction is largely due to the high postoperative complications rate after thyroid surgery plus lymph node dissection performed in centers with limited experience. Indeed, in high-volume centers, it seems that prophylactic central lymph node dissection doesn’t influence the postoperative complications rate, as confirm our results and other published papers [67,68,73,74,75].

Therefore, a careful preoperative evaluation in cN0 patients is mandatory to select the most effective surgical treatment more appropriately. In the present study, we aimed to identify predictors of CLNM in PTMC to preoperatively assess the clinical and US features associated with the theme.

We found several useful and independent risk factors predicting CLNM in PTMC patients, such as male gender, younger age, and sub-capsular tumor localization. Combinational use of these findings together with preoperative ultrasonography might help clinicians to precisely estimate the probability of the existence of CLNM in PTMC patients, which is helpful to define proper individualized surgical planning. Based on these preoperative features of PTMCs, tailored treatment strategies including active surveillance, ultrasound-guided minimally invasive treatments, lobectomy, or total thyroidectomy with or without central lymph node dissection should be carefully considered [25,26]. Central compartment lymph node dissection can be achieved with low morbidity in experienced hands, and with a very low rate of complications such as hypoparathyroidism and recurrent laryngeal nerve injury [68]. For patients with papillary thyroid micro-carcinoma, the presence of these factors should be considered as risks for CLNM, and, in these cases, a prophylactic routine central compartment (level VI) neck dissection should be planned. Our multivariate analysis identifies significant factors independent of each other to predict CLNM. Their significance in combination with different analyses may be the subject of future studies to achieve the conclusion of Liu C et al. (2021): “the dissection should be performed especially for cases where a combination of risk factors is present” [24].

Some limitations of our study should be considered, such as the monocentric nature and retrospective analysis with potential selection, and observational and data collection biases. Another limitation is the small sample size. On the other hand, this is a cohort study with accurate medical records and without patients with missing data.

## 5. Conclusions

In summary, the findings of our study suggest that central compartment lymph node metastases are present in a non-negligible number of cases in patients with papillary thyroid micro-carcinoma undergoing surgical resection. Male gender, younger age, and histopathological characteristics, such as a tumor sub-capsular localization, might be predictors of CLNM. Our findings may help clinicians and surgeons in selecting patients suitable for either conservative or aggressive approaches, ranging from clinical observation with active surveillance, to ultrasound-guided minimally invasive treatments, [25] or to surgical treatment with or without prophylactic CLND. Routine prophylactic central lymph node dissection at the time of thyroid surgery may be recommended in PTMC patients with risk factors for CLNM. More studies are needed to validate our preliminary results and provide an effective tool for predicting CLNM in PTMC patients and defining their tailored treatment in the future.

## Figures and Tables

**Table 1:** Baseline and clinicopathologic characteristics of 195 patients treated with surgery for PTMC.

Overall Selected Patients	(*n* = 195)
Age, years (mean ± SD)	48 ± 13
Gender	Female: 163 (84%) Male: 32 (16%)
Central LN metastases	52 (27%)
Distant metastases	0 (0%)
Multifocality	56 (29%)
Follicular variant subtype	52 (27%)
Extent of surgery	TT: 139 (71%) TL: 56 (29%)
Sub-capsular localization	66 (34%)

PTMC: papillary thyroid micro-carcinoma; SD: standard deviation; LN: lymph nodes; TT: total thyroidectomy; TL: thyroid lobectomy.

**Table 2:** The clinical and pathological characteristics of central lymph nodes of 195 patients treated with surgery for PTMC.

	CLN Positive (*n* = 52, 27%)	CLN Negative (*n* = 143, 73%)	*p*-Value
Age, years (mean ± SD)	45 ± 13	50 ± 12	0.012
Gender	Female: 37 (71%) Male: 15 (29%)	Female: 126 (88%) Male: 17 (12%)	0.008
Sub-capsular localization	25 (48%)	41 (28%)	0.01
Multifocality	20 (38%)	36 (25%)	0.07
Follicular variant subtype	10 (19%)	42 (29%)	0.2

PTMC: papillary thyroid micro-carcinoma; CLN: central lymph nodes.

**Table 3:** Multivariate analysis of risk factors associated with central lymph node metastases in 195 patients treated with surgery for PTMC.

Variables	Odds Ratio [95% CI]	*p*-Value
Age	0.96 [0.94–0.99]	0.018
Gender	2.86 [1.24–6.6]	0.014
Sub-capsular localization	2.03 [1.01–4.07]	0.04
Multifocality	1.85 [0.9–3.79]	0.09
Follicular variant subtype	0.7 [0.3–1.65]	0.43

## Data Availability

The data presented in this study are available on request from the corresponding author.
